# Exploring the Dose–Response Relationship Between Alcohol Consumption and Harm to Others in Australia

**DOI:** 10.1111/dar.70226

**Published:** 2026-08-07

**Authors:** Koen Smit, Heng Jiang, Katherine J. Karriker‐Jaffe, Anne‐Marie Laslett

**Affiliations:** ^1^ Centre for Alcohol Policy Research, School of Psychology and Public Health La Trobe University Melbourne Australia; ^2^ Melbourne School of Population and Global Health University of Melbourne Melbourne Australia; ^3^ Department of Public Health, School of Psychology and Public Health La Trobe University Melbourne Australia; ^4^ Center for Behavioral Health & Survey Methods RTI International Research Triangle Park North Carolina USA; ^5^ Care Economy Research Institute La Trobe University Melbourne Australia

**Keywords:** alcohol consumption, alcohol's harms to others, dose–response relationship, heavy episodic drinking, socio‐demographic factors

## Abstract

**Introduction:**

Alcohol consumption often leads to harm to individuals other than the drinker. Yet, few studies have quantified an exposure‐response gradient using “nominated” drinkers' alcohol volume and their heavy episodic drinking (HED) frequency to predict harm‐type burden and overall severity among people harmed by someone else's alcohol use. We examined these dose–response relationships across sociodemographic groups.

**Methods:**

Data were drawn from the cross‐sectional Australian 2021 Alcohol's Harm to Others (AHTO) Survey. A total of 2574 adults (18+ years) participated, of whom 554 reported harm from another person's drinking in the past 12 months. Respondents identified the person who harmed them the most (the “nominated drinker”) and reported on the person's weekly alcohol volume and frequency of HED. Harms experienced included an overall harm score (0–10 scale) and a count of 14 specific harms.

**Results:**

There was a significant positive dose–response relationship between the nominated drinker's alcohol consumption (both weekly volume and frequency of HED) and the degree of harm experienced by respondents. Although subgroup analyses suggested stronger effects among women, younger respondents, and when the drinker was a (former) partner, these differences were not statistically significant.

**Discussion and Conclusions:**

Greater alcohol consumption and more frequent HED are associated with more severe harms to others, regardless of respondents' sociodemographic background. A clear dose–response relationship exists between alcohol consumption by a known drinker and harm to others. The dose–response gradient was stronger at higher exposure levels, suggesting that reducing very frequent heavy drinking may yield disproportionately larger reductions in AHTO burden.

## 1 Introduction

In Australia, per capita alcohol consumption has increased since 2019 [1], and alcohol use continues to be a significant concern for public health [1, 2], as drinking is linked to over 200 health conditions and increased healthcare costs [3]. Alcohol also has common and serious consequences for people affected by others' drinking, with these harms described as alcohol's harm to others (AHTO). AHTO is widespread and affects people of all ages, genders and societies [4, 5, 6]. AHTO includes: social problems; financial stress; disruptions in daily life due to caring for people who drink harmfully; and diminished health related quality of life [4, 5, 7, 8, 9]. Despite increased awareness of AHTO, we know little about the patterns and levels of drinking of alcohol consumers that are associated with increasing amounts and severity of harms experienced by others. Therefore, this study aims to examine the dose–response relationship between the drinking of the person causing harm (dose) and the harms experienced by the individual (response).

A dose–response association has proven valuable for quantifying negative consequences from alcohol use. For example, studies have identified a dose–response relationship in the frequency of common alcohol‐related problems (e.g., absenteeism from work, involvement in physical fights, injury risk) that are proportional to annual volume of consumption of the person drinking [10, 11]. Similarly, higher alcohol use was associated with increased risk of aggressive incidents among psychiatric patients [12]. While the concept of a dose–response relationship between alcohol use and harm is well established, relatively few studies examine this in the context of AHTO. In this study, we use the term dose–response in the epidemiologic sense of a graded association between level of alcohol exposure and harm outcomes. The “dose” refers to the nominated drinker's alcohol consumption level and the “response” to the severity of harm experienced by the individual. A dose–response may arise because heavier alcohol consumption volumes increase exposure opportunities, while frequent heavy episodic drinking (HED) decreases intoxication‐related control and increases conflict escalation. Both may amplify the likelihood and breadth of harms. Rehm and colleagues argue that documented dose–response relationships provide vital information for policy and prevention measures to reduce harmful alcohol use [13]. For example, if dose–response relationships are demonstrated, especially if they are found to be steep or exponential, prevention measures targeting these specific levels of drinking should be more (cost‐)effective in preventing alcohol‐related consequences.

In addition to examining the general dose–response relationship between alcohol use and AHTO, disparities in the dose–response relationship as a function of socio‐demographic indicators, particularly gender, should be examined. More harm from others' drinking is borne by women than by men [6, 14, 15]. A recent literature review showed that the influence of alcohol is greater in men‐to‐women partner violence than vice versa [16], while a meta‐analysis showed that regardless of the sex of the perpetrator, the association between drug misuse and aggression is bigger for women who experience harm than for men [17]. Similarly, relationship context may influence the slope of harm exposure, but evidence about the different relationship types responsible for alcohol‐related harms by the drinkers in one's environment is scarce. While one study indicated that AHTO is most frequently perpetrated by friends [18], specific harms such as physical injuries might be more commonly perpetrated by partners or ex‐partners. Finally, structural factors may influence the dose–response relationships. Younger people are more likely to be involved in heavy episodic or “binge” drinking [19]. Additionally, individuals in socio‐economically disadvantaged areas are disproportionally affected by alcohol use in general, as well as being disproportionally harmed per litre of alcohol consumed [20, 21]. Further, both individual‐level poverty [22] and area‐level disadvantage [23] are associated with increased risk of AHTO. This study also explores whether the dose–response relationship varies across respondent gender, age, socioeconomic status and/or geographic area (rural vs. urban).

In summary, this study aimed to investigate the dose–response relationship for the alcohol use of the nominated drinker (i.e., individual whose drinking was nominated as most harmful) in relation to the degree of AHTO experienced by an individual (i.e., the person experiencing harm). We expected a linear association, potentially with a steeper gradient at higher levels of HED frequency and greater weekly drinking volume. Our specific objectives are as follows:

- Aim 1. To characterise nominated drinkers based on gender, age, drinking behaviour and relationship to harmed individuals.
- Aim 2. To investigate the overall dose–response relationship between alcohol use of the nominated drinker and the degree of harm experienced by the harmed individual.
- Aim 3. To explore whether the dose–response relationship differs by respondent gender, relationship type, age, rurality and socio‐economic characteristics.

## 2 Methods

### 2.1 Study Design and Procedure

The current study used weighted cross‐sectional data from the 2021 Australian Alcohol's Harms to Others Survey. Data were collected from 2574 Australian adults using a combination of a national random‐digit dialled Australian mobile phone sample (*n* = 1000) and the national Life in Australia panel survey (*n* = 1574). Sampling procedures were designed to achieve demographic representativeness of the Australian population. The combined response rate was low (6.1%). More information on the study design and data collection protocols is available elsewhere [5, 6, 24]. The survey took about 30 min to complete. Random‐digit dialled participants were encouraged to participate by offering prizes, and respondents in the panel survey received a voucher of AUD$15 to compensate them for their time.

The analytic sample consists of the subgroup of Australian adults who indicated having one (*n* = 369) or more (*n* = 201) people who drink heavily in their life in 2021; all other respondents were excluded from the analysis given the current study's focus on characteristics and harms from one drinker that was nominated by each respondent (nominated drinker). The study is reported in accordance with the STROBE guidelines [25]. The analysis was not pre‐registered; therefore, the results should be considered exploratory. Ethics approval was obtained from La Trobe University's Human Ethics Research Committee (HEC20518).

### 2.2 Measures

#### 2.2.1 The Nominated Drinker

Respondents were asked whether “at any time in the last 12 months, has there been a member of the household, relative, partner, co‐worker, or other person you know well who you would consider to be fairly heavy drinker or someone who drinks a lot sometimes?” and subsequently, those with a heavy drinker in their life were asked “would you say their drinking negatively affected you in some way in the last 12 months?” Through these questions, respondents identified one or more known heavy drinkers whose drinking negatively affected them over the past year. After this, respondents were further asked “thinking about all of these people, overall, whose drinking has *most* negatively affected you in the last 12 months?” This item identified a “nominated drinker” whose drinking had most negatively affected the respondent. People with a “non‐harmful” heavy drinker in their life were excluded from the analysis.

#### 2.2.2 Demographics

The respondent's gender and age were recorded, as well as the gender and age of the nominated drinker (for descriptive purposes only). Based on the respondent's postcode, we included information on their geographical area of residence (state capital city vs. rest of state) and the Socio‐Economic Index for Areas (SEIFA), which was categorised into two levels: 1 (disadvantaged, lowest two quintiles) and 2 (advantaged, highest three quintiles) [26].

#### 2.2.3 Relationship Type

Respondents were asked about their relationship with the nominated drinker. They could choose from several response options, including spouse or partner living with you, immediate family member not in the household, current or ex‐boyfriend/girlfriend, friends, colleagues and others. These options were categorised into: (0) family; (1) current and ex‐partners; and (2) friends, colleagues and others.

#### 2.2.4 Average Weekly Alcohol Volume

Respondents were asked how often the nominated drinker (and the respondents themselves) consumed alcohol and how much alcohol is typically consumed. Note that the exposure measures captured respondents' perceptions of the nominated drinker's alcohol use rather than objective consumption. Average weekly alcohol volume was calculated using the frequency and quantity of alcohol use. Frequency was measured with the question “In the last 12 months, how often did (the nominated drinker) have an alcoholic drink of any kind?”. Responses ranged from “every day” to “never drank alcohol”. Quantity was measured with the question: “On a day that the drinker (you nominated) has an alcoholic drink, how many standard drinks do you think they would usually have?” The answers ranged from (1) 20 or more to (7) less than 1 standard drink per day, and they were recoded such that higher values indicate higher volume consumed per drinking day. The average weekly volume in standard drinks (based on 10 g ethanol/drink) was calculated and categorised as 1–4, 5–9, 10–19, 20–29, 30–39, 40–54, 55–69, 70+ standard drinks (SD) per week. A similar measure was created to describe the respondent's own weekly alcohol use. Categories were determined to achieve approximately equal cell sizes and ensure sufficient sample sizes within each subgroup. Sensitivity analyses using continuous alcohol consumption measures produced broadly similar dose–response relationships (results not shown).

#### 2.2.5 Heavy Episodic Drinking

Respondents were asked how often the nominated drinker consumed 5+ standard drinks (i.e., ≥ 50 g of ethanol) on a single occasion in the last year. Responses ranged from (1) every day to (8) not in the last 12 months, with a category for never (9). HED of the nominated drinker was defined as individuals who drank 5+ standard drinks on an occasion at least once a month up to every day (HED‐*nd*), Those who drank 5+ standard drinks less than once a month or not at all in the past year were included in a separate category [27]. A similar measure was created to describe the respondent's own heavy episodic drinking (HED‐*r*).

#### 2.2.6 Harm Outcomes

Respondents were asked 14 questions about the harms they experienced over the last 12 months because of others' alcohol use. Harms included felt threatened or afraid; serious argument; emotionally hurt or neglected; physically hurt by them; person failed to do something they were being counted on to do; had to stop seeing them; forced or pressured into sex or something sexual; negatively affected in a social occasion; financial trouble; family problems or relationship difficulties; involved in a traffic accident; house or other property damaged (please see full list of harm items in Table S1). Using a single item, respondents were asked to rate on a scale from 0 to 10 the adverse effects they had experienced because of others' drinking. These harm scores represent the level of severity of harm experienced. Harms also were used as a count variable to represent the number of distinct harm types reported, and these were used in supplemental analyses (see Tables S1–S6). The severity outcome was used as the primary measure of harm intensity, while the count of harm types endorsed captures breadth of burden but weights all harm types equally.

### 2.3 Analyses

First, we used weighted descriptive statistics to examine the characteristics of respondents and their nominated drinkers. Second, we used generalised linear model (GLM) with a Gamma distribution with a log link function (log link function is a default for Gamma family in GLM) to assess associations between the nominated drinker's alcohol consumption patterns (weekly volume, HED frequency) and harm scores and the degree of harm experienced by respondents in the past 12 months (analysed in separate models), controlling for the respondent's age, gender, SEIFA, number of nominated drinkers and relationship characteristics (i.e., relationship type to the most harmful nominated drinker). The primary models used complete‐case analysis; sample sizes vary by exposure due to missing nominated drinker volume (*n* = 470) and HED (*n* = 536).

The outcome variables (harm scores and number of harm types endorsed) are strictly positive and right‐skewed, thus GLM with a Gamma distribution with a log link function ensures that predictions are positive and allows multiplicative interpretations. Model coefficients are on the log scale (rate ratios); we present results primarily as predicted outcomes for interpretability. “Margins” function was used to predict values of the outcome variable at different values of weekly alcohol consumption or HED for various subgroups. A post‐estimation command “*marginsplot*” was used to plot the dose–response relationship (HED/level of weekly consumption vs. predicted harm) separately for each subgroup. These methods have been used in our previous studies [28].

To test the overall dose–response relationship, we used Wald tests to demonstrate whether all levels of HED or weekly alcohol consumption have the same effects compared to the reference category (reference groups are “Less often” in HED and “1–4 SDs/week” in Weekly drinking volume). Wald tests (using the *testparm* command) were applied to test significance of subgroup differences in the dose–response relationship based on gender and age. We then used the margins function to plot the dose–response relationships between nominated drinkers' alcohol consumption and degree of harm experienced by respondents across the overall sample and within various subgroups of respondent characteristics. Subgroup differences were discussed based on the observed relationships, as well as significant Wald tests (*p* < 0.05). All analyses were performed using Stata 18 [29].

## 3 Results

### 3.1 Characteristics of the Sample of Nominated Drinkers

Table 1 presents the weighted characteristics of the nominated drinkers and of the respondents. Of the 552 respondents with information on a nominated heavy drinker, we have estimated information on the nominated drinker's average weekly alcohol volume for 470 (85.1%), and on HED for 536 (97.1%). Data were complete for the respondent's own weekly alcohol volume, and almost complete for their own HED (*n* = 551, 99.8%).

**TABLE 1:** Descriptive statistics of nominated drinkers and respondents by sociodemographic and drinking characteristics.

	Nominated drinkers		Respondents	
	*N*	Weighted %	*N*	Weighted %
Gender				
Male	355	66.3%	213	42.9%
Female	197	33.7%	334	57.1%
Age category, years				
18–34	69	14.9%	126	30.3%
35–64	350	63.2%	307	55.8%
65+	132	21.9%	119	13.9%
SEIFA				
1 (Disadvantaged)	—	—	203	40.9%
2 (Advantaged)	—	—	343	59.1%
Geographic area^a^				
Urban (capital city)	—	—	337	59.0%
Rural (rest of state)	—	—	209	41.0%
Heavy episodic drinking				
Less often	40	6.4%	378	66.1%
Less than 1 day per week	51	3.3%	104	18.3%
1–2 days per week	117	5.7	50	8.8%
3–4 days per week	94	14.4	21	4.1%
5–6 days per week	78	17.8%	7	1.0%
Everyday	156	31.1%	8	1.8%
Average weekly alcohol volume				
1–4 SDs per week	18	4.4%	271	60.4%
5–9 SDs per week	27	6.8%	101	19.8%
10–19 SDs per week	98	21.3%	63	11.7%
20–29 SDs per week	57	9.7%	12	2.7%
30–39 SDs per week	88	16.1%	12	2.5%
40–54 SDs per week	30	6.0%	2	0.1%
55–69 SDs per week	76	17.4%	4	0.1%
70 + SDs per week	76	18.5%	3	0.1%

Relation between nominated drinker and respondent	*N*	Weighted %
(Ex‐)partners	109	19.8%
Other family	245	42.2%
Friends and others	197	38.1%

^a^ SEIFA (socioeconomic status) and geographic area (urban/rural) were differently distributed among the sample from what would be expected by chance (chi‐square test, *χ* ^2^ (1) = 59.37, *p* < 0.001). Results for SEIFA and rural or urban settings are therefore presented separately in the Supporting Information. Abbreviations: SD, standard drink; SEIFA, Socio‐Economic Index for Areas.

Most of the nominated drinkers were men, but a third were women. In contrast, the majority of respondents were women (about 57%). About two‐thirds (63%) of nominated drinkers were between 35 and 64 years of age, 15% were between 18 and 34 years of age, and 22% were aged 65 years or older. Similar age distributions were observed among the respondents. About 42% of nominated drinkers were family members, a third were friends or others (e.g., co‐workers), and about 20% were current or ex‐partners. Lastly, most (84%) nominated drinkers engaged in HED more than one time per week. By contrast, respondents engaged in HED less frequently and consumed less alcohol than the nominated drinkers.

### 3.2 Dose–Response Relationships

**FIGURE 1:**
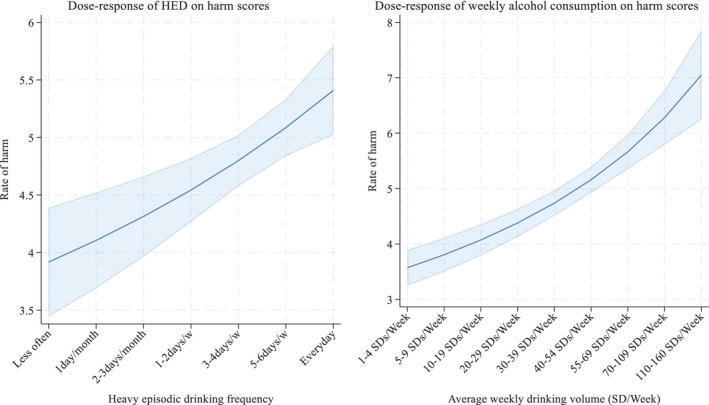
Dose–response relationship of alcohol consumption (frequency of heavy episodic drinking [HED] and mean weekly consumption volume) with harm scores (severity levels) (margins prediction were presented in Supporting Information).

Figure 1 shows positive and accelerated dose–response relationships of frequency of the nominated drinker's HED (*χ* ^2^ (1) = 6.72, *p* < 0.001) and average weekly alcohol volume (*χ* ^2^ (1) = 36.86, *p* < 0.001) with severity of harm experienced, suggesting that the higher the estimated alcohol consumption of the nominated drinker, the higher the levels of harm caused to respondents (Table 2). Shaded regions represent 95% confidence intervals around the predicted means. Similar relationships were found for the harm types endorsed for both HED (*χ* ^2^ (1) = 11.68, *p* < 0.001) and volume (*χ* ^2^ (1) = 52.10, *p* < 0.001) (see Figures S1–S6).

**TABLE 2:** Weighted model outputs for rate of harm (harm score) experienced and alcohol consumption (HED and weekly drinking volume).

Variables	Harm scores and HED			Harm scores and weekly drinking volume		
	Coeff.	*p* > *z*	95% CI	Coeff.	*p* > *z*	95% CI
Frequency of HED (categorical from low to high)	0.049	0.010	0.012, 0.086			
Weekly drinking volume (categorical from low to high)				0.089	0.000	0.060, 0.118
Gender (ref: Male)						
Female	0.138	0.023	0.019, 0.257	0.151	0.013	0.032, 0.271
Age, years (ref: 18–34)						
35–64	−0.073	0.307	−0.212, 0.067	−0.093	0.173	−0.227, 0.041
65+	0.073	0.411	−0.102, 0.248	0.097	0.293	−0.084, 0.277
SEIFA (ref: quintile 1)						
Quintile 2	0.005	0.963	−0.185, 0.194	0.105	0.282	−0.086, 0.296
Quintile 3	0.016	0.863	−0.169, 0.202	0.123	0.192	−0.062, 0.307
Quintile 4	−0.006	0.949	−0.195, 0.182	0.056	0.542	−0.126, 0.238
Quintile 5 (highest)	−0.032	0.766	−0.243, 0.179	−0.011	0.915	−0.221, 0.198
Number of drinkers (ref: 1)						
≥ 2 drinkers	0.087	0.177	−0.039, 0.213	0.024	0.702	−0.100, 0.148
Relationship (ref: Partner/ex)						
Family	0.035	0.696	−0.143, 0.214	0.044	0.608	−0.125, 0.214
Friends and others	−0.048	0.584	−0.220, 0.124	−0.009	0.910	−0.169, 0.151
Region (ref: Capital city)						
Rest of state	−0.013	0.839	−0.139, 0.113	−0.059	0.361	−0.186, 0.068

*Note:* HED frequency and weekly alcohol volume categories were treated as continuous variables in the regression models.

Abbreviations: CI, confidence interval; HED, heavy episodic drinking; SEIFA, Socio‐Economic Index for Areas.

### 3.3 Subgroup Analyses

**FIGURE 2:**
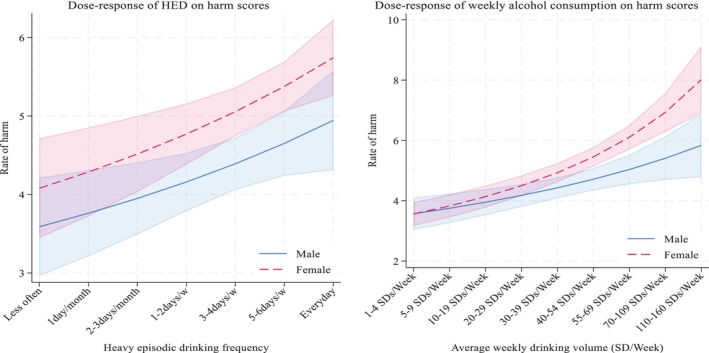
Dose–response relationship between alcohol consumption [frequency of heavy episodic drinking (left panel) and weekly consumption volume (right panel)] and rate of harms experienced by gender of respondents.

Figure 2 shows a similar dose–relationship between the nominated drinker's alcohol consumption and the degree of harm experienced by men and women due to others' drinking. At higher levels of consumption (doses in standard drinks per week), but not higher frequency of heavy episodic drinking, women seem to be more likely to report a greater level of harms than men. However, Wald tests indicated that the subgroup differences were non‐significant for both HED (*χ* ^2^ (1) = 0.02, *p* = 0.891) and level of consumption (*χ* ^2^ (1) = 0.15, *p* = 0.225). Full regression model results of the dose–response relationships can be found in Tables S2–S5.

**FIGURE 3:**
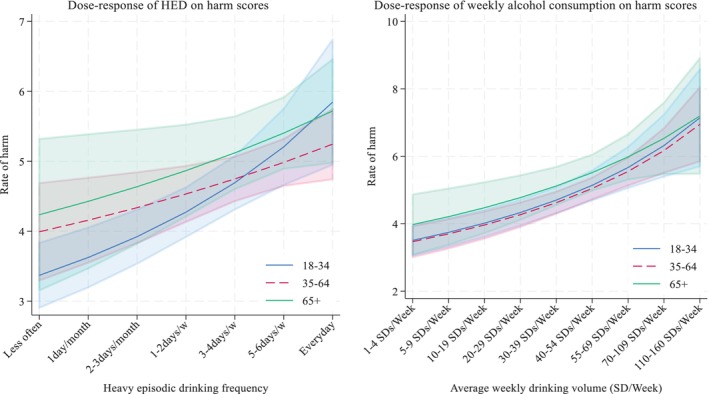
Dose–response relationship between alcohol consumption [frequency of heavy episodic drinking (HED, left panel) and weekly consumption volume (right panel)] and rate of harms by three age groups of respondents.

In terms of age groups, accelerated dose–response relationships were found between the nominated drinker's average weekly alcohol volume and the harm outcomes for respondents in younger age groups (18–34 years) compared with the older age group (65+ years) (Figure 3). However, Wald tests indicated that the subgroup differences were non‐significant for both HED (*χ* ^2^ (2) = 3.27, *p* = 0.195) and level of weekly alcohol consumption (*χ* ^2^ (1) = 0.49, *p* = 0.783).

**FIGURE 4:**
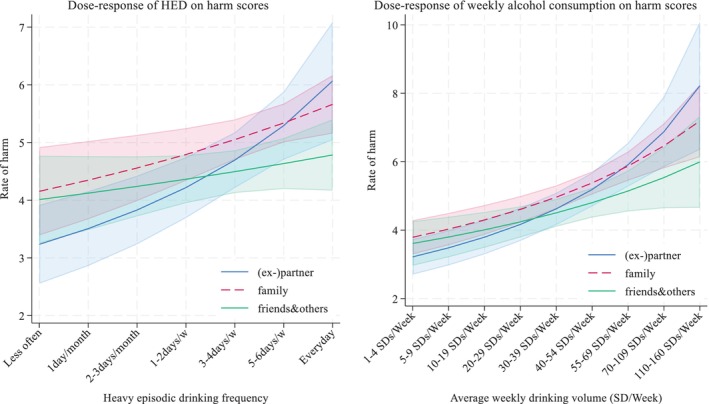
Dose–response relationship between alcohol consumption [frequency of heavy episodic drinking (HED, left panel) and weekly consumption volume (right panel)] and rate/severity of harm by the relationship with the most harmful heavy drinker.

When the relationship between the respondent and the nominated drinker is considered, the HED and weekly alcohol consumption of partners and ex‐partners is more strongly and positively associated with severity of harm experienced by the respondent, compared with drinking of family members or friends/others, indicating a stronger dose–response relationship for partners and ex‐partners than other relationship types (Figure 4). However, Wald tests indicated that these subgroup differences were non‐significant for both HED (*χ* ^2^ (2) = 4.28, *p* = 0.118) and volume (*χ* ^2^ (2) = 2.96, *p* = 0.228).

Overall, the dose–response relationships for the severity of harms experienced (y‐axis) in the total sample, as well as by gender, age and relationship type, look very similar to the figures based on the number of types of harms endorsed, presented as Supporting Information (Figures S2–S4). Additionally, dose–response relationships differed neither visibly nor significantly (*p*‐value > 0.05) across geographic areas (urban vs. rural) or by low versus high SEIFA categories (see Figures S5 and S6) in any of the analyses.

## 4 Discussion

The current study examined the dose–response relationship between the alcohol use of the nominated drinker and respondents' experiences of severity and the number of types of harms from this person's drinking. A significant positive and accelerated dose–response relationship was identified between average weekly alcohol volume and the number and severity of the harms from others' drinking. A similar positive dose–response relationship was observed for the frequency of heavy episodic drinking. These dose–response relationships were not significantly different across genders, age, relationship types, and regional and socioeconomic subgroups.

The observed age and gender similarities in the dose–response relationship between alcohol consumption and harms to others indicate that the relationship spans demographic groups. However, it is important to note that we are comparing harms experienced *by* men and women regardless of the nominated drinker's gender. It might be expected that both women and men similarly report on the dose–response, with this relationship primarily applying to men's drinking, as two‐thirds of the nominated drinkers were men. This analysis does not explore whether the likelihood of causing harm to others varies across the gender of the person consuming alcohol, but rather it reports on men and women's experiences of harm as a nominated harmful drinker's level of alcohol consumption increases. Future analyses should include the gender of the nominated drinker as well as capturing alcohol consumption of people who use alcohol heavily but do not cause harm to others. Future research should also increase the sample size to test for gendered differences and explore mechanisms (e.g., social norms, stigma and social interaction) that might contribute to explanations of the dose–response relationship documented here.

Regardless of relationship type, the dose–response relationship seems to hold. While previous research highlights the important role of partners and ex‐partners in alcohol‐related harms [30, 31, 32], the current findings suggest that the risk of experiencing harms due to others' drinking similarly increases with the amount consumed by the nominated drinker, independent of relationship type. This trend underscores the relevance of targeting harmful drinking patterns in interventions, without only focusing on intimate relationships [33, 34]. Future research with larger samples should clarify whether the gender of the nominated harmful drinker moderates the effects of specific relationship contexts on the degree of harm experienced.

In addition, our study did not observe significant differences in dose–response relationships based on SEIFA levels or metropolitan versus more rural location. Although previous research has highlighted that those in disadvantaged areas are disproportionally affected by alcohol [20], on both the individual level and area‐level [22, 23], the current results indicate that the dose–response relationship remained consistent across the two socioeconomic contexts we studied. More detailed measures of socio‐demographic disadvantage on a larger sample would increase our capacity to evaluate such effects. Potential explanations for a lack of evidence suggesting socioeconomic differences include a relatively high harm baseline: Disadvantaged areas may experience higher levels of harms due to higher levels of poverty and stress. Tolerance again may come into play, with respondents who have experienced multiple types of disadvantage being more likely see some specific harms from others drinking as almost normative, thereby resulting in less perception of certain types of harms from others' drinking as necessarily harmful. As a result, harms may be underreported in areas with high harm exposure. The absence of an accelerated dose–response relationship does not negate the severity of harms in disadvantaged individuals or areas. Instead, it shows the need for addressing baseline harm levels, as well as drinking behaviours that contribute to alcohol‐related harms. Finally, it should be noted that subgroup patterns by gender or geographic area should be interpreted descriptively, as differences may also reflect variation in drinking cultures, harm prevalence or nomination processes rather than true effect modification.

### 4.1 Limitations and Future Research

Although this study is based on a substantial survey sample, some limitations should be acknowledged. First, while analytic weights improved demographic alignment, one limitation of this survey is its low response rate, for details see [6, 24]. However, response rates are in line with survey trends [5, 35], which were found to be only marginally less accurate compared to studies with higher response rates [35]. Nonetheless, potential selection effects should be considered when interpreting the results. In addition, it should be mentioned that when more than one drinker was identified, only the characteristics of the nominated drinker were collected. It was not possible to collect data on all drinkers who had affected the respondent. Also, people with a heavy drinker in their life who did not experience any harm that they attributed to this drinker are excluded from the analysis. Future studies are needed to ascertain relationships with non‐harmful heavy drinkers to extend our understanding of the dose–response relationship to include people who have not been harmed recently by someone else's alcohol use.

Second, another limitation of this study is that it relies on the perspective of a single individual nominating the person whose drinking has most harmed them in the last 12 months (“nominated drinkers”). These proxy reports may be prone to random error and systematic bias. Moreover, it may result in underestimating the dose–response relationship with harms to others, as specific drinkers may have caused harm to multiple individuals. Accordingly, subgroup patterns by gender or geographic area should be interpreted descriptively and not as evidence of effect modification. As subgroup estimates were imprecise (wide confidence intervals), null interaction tests should not be interpreted as evidence of no heterogeneity. Furthermore, as the nominated drinker was identified as the person causing the most harm, the dose–response relationship estimated here applies specifically to this selected group and may not generalise to the full range of drinkers in the population. In the future, more work could be done on the characteristics of the nominated drinker(s) and how this affects the dose–response relationship. Future research should also examine intoxication level and drinking frequency as separate dimensions of alcohol exposure, to better understand which aspects of drinking patterns are most strongly associated with specific types of harm to others.

Another limitation of the present study is the small cell sizes, which limited our capacity to study the gender of both the respondent and the nominated drinker, or interactions between relationship type and gender. This is a critical gap, as both relationship context and gender dynamics have been shown to influence alcohol‐related harms [6]. Lastly, given the cross‐sectional design, results represent associations and cannot establish causality. Reverse causation and shared confounding are possible. Longitudinal designs that track changes in drinking within the same nominated individual over time could reduce selection bias and strengthen causal inference, although such approaches must carefully address ethical considerations related to collecting information about third parties.

Of note, the current study was undertaken in 2021 during the COVID‐19 pandemic. Licensed premise closures and social distancing policies affected the ways in which Australians consumed alcohol in 2021 [36]. Lockdown restrictions limited contacts with friends and strangers (outside the household), whereas contact with household members (e.g., partners and family) increased. However, take away alcohol sales were not restricted when Australia was locked down during the pandemic, and this has been associated with increases in domestic violence [37]. Other studies also reported increases in domestic violence during the pandemic [38, 39, 40]. Our study suggests that people who were harmed by someone else's alcohol use were at greater risk of harms as the other person's alcohol consumption and heavy drinking increased. Altogether, the influence of COVID‐19 on Australian adults' drinking behaviour and AHTO should be considered when interpreting the results of this study. This unique social context means these results need to be interpreted cautiously if extrapolated beyond the pandemic.

### 4.2 Practical and Policy Implications

The current study underlines the need to reduce average weekly alcohol volumes and heavy episodic drinking to reduce harm. Predicted harm outcomes differed substantially across categories of nominated drinker HED frequency. For example, respondents whose nominated drinker engaged in daily HED reported higher predicted harm counts and severity scores than those whose nominated drinker did so only monthly.

These findings could inform public health campaigns that each drink increases the risk of harm not only to oneself but to others around you. Future research is needed to increase understanding of the profiles of people who drink in ways that harm others. This information might help healthcare professionals identify drinking thresholds and contexts where people are at increased risk of experiencing harm from someone else's alcohol use. Simultaneously, this knowledge could inform behaviour change interventions directed towards individuals at risk of causing harm to others.

## Author Contributions

Each author certifies that their contribution to this work meets the standards of the International Committee of Medical Journal Editors.

## Funding

The survey and associated research were funded by the Australian Research Council linkage project (LP190100698) and the Foundation for Alcohol Research and Education. K.S. was supported by an Australian Rechabite Fund grant and LP190100698. H.J. was supported by an Australian Research Council Discovery Project Grant (DP200101781). A.‐M.L. and the AHTO team were supported by National Health and Medical Research Council (GNT2016706). Support for K.J.K.‐J. was provided by a research grant from the U.S. National Institutes of Health/National Institute on Alcohol Abuse and Alcoholism (R01AA029001). The content is solely the responsibility of the authors and does not necessarily represent the official view of the National Institutes of Health.

## Conflicts of Interest

The authors declare no conflicts of interest.

## Supporting information


**Table S1:** Alcohol's harms to others items.
**Table S2:** Model outputs for number of types of harm (harm items) experienced and frequency of heavy episodic drinking (HED).
**Table S3:** Model outputs for number of harm (harm items) experienced and weekly alcohol consumption.
**Figure S1:** Dose–response relationship of alcohol consumption (mean weekly consumption [right panel] and frequency of heavy episodic drinking [left panel]) with the number of harms experienced.
**Figure S2:** Dose–response relationship between alcohol consumption [heavy episodic drinking (left panel) and frequency of weekly consumption volume (right panel)] and number of harms experienced by gender of respondents.
**Figure S3:** Dose–response relationship between alcohol consumption [heavy episodic drinking (left panel) and weekly consumption volume (right panel) and frequency] by three age groups of respondents.
**Figure S4:** Dose–response relationship between alcohol consumption [weekly consumption volume (left panel) and frequency of heavy episodic drinking (right panel)] by the relationship with the most harmful heavy drinker.
**Figure S5:** Dose–response relationship between alcohol consumption [frequency of heavy episodic drinking (panels A (*χ* ^2^ (1) = 0.02, *p* = 0.900) and C (*χ* ^2^ (1) = 0.00, *p* = 0.975)) and weekly consumption volume (panels B (*χ* ^2^ (1) = 0.08, *p* = 0.778) and D (*χ* ^2^ (1) = 0.17, *p* = 0.684))] and alcohol‐related harm experience [harm rates (panels A and B) and number of harm items experienced (panels C and D)] by the socioeconomic status (SEIFA index) of respondents.
**Figure S6:** Dose–response relationship between alcohol consumption [frequency of heavy episodic drinking (panels A and C) and weekly consumption volume (panels B and D)] and alcohol‐related harm experience [harm rates (panels A (C (*χ* ^2^ (1) = 1.54, *p* = 0.215)) and B (C (*χ* ^2^ (1) = 0.10, *p* = 0.750)) and number of harm items experienced (panels C (*χ* ^2^ (1) = 2.98. *p* = 0.084) and *D* (*χ* ^2^ (1) = 3.53. *p* = 0.060))] by the region (urban vs. rural) of respondents.

## Data Availability

The data that support the findings of this study are available from the corresponding author upon reasonable request.
