# Supplementary material for: Exploring the Dose–Response Relationship Between Alcohol Consumption and Harm to Others in Australia
Source: Drug Alcohol Rev. 2026 Aug 7;45(6):e70226. doi: 10.1111/dar.70226 (PMC13449624; doi:10.1111/dar.70226)
Supplement: Supplementary file 1 [file DAR-45-0-s001.docx]

**Supplementary Tables and Figures.**

| **Table S1**  Alcohol’s Harms to Others Items | | | | | | |
| --- | --- | --- | --- | --- | --- | --- |
| Variable code | Variable name | Content |  | %yes | %yes | %yes |
|  |  |  | N | total | men | women |
|  |  |  |  |  |  |  |
| E1a | names | Were you called names or otherwise insulted? | 568 | 38.20% | 36.16% | 42.64% |
| E1b | threat | Did you feel threatened or afraid because of the drinking of any of the drinkers you have identified? | 566 | 21.91% | 23.01% | 20.81% |
| E1c | argument | Did you have a serious argument that did NOT include physical violence because of their drinking? | 568 | 43.13% | 42.54% | 43.37% |
| E1d | emohurt | Were you emotionally hurt or neglected because of their drinking? | 563 | 55.77% | 51.99% | 61.34% |
| E1e | physhurt | Were you physically hurt by any of these drinkers because of their drinking? (e.g., pushed or shoved/harmed physically?) | 567 | 7.41% | 7.06% | 7.65% |
| E1f | responsib | Did they fail to do something they were being counted on to do because of their drinking? | 561 | 56.68% | 56.61% | 56.63% |
| E1g | seeing | Did you have to stop seeing any of these drinkers because of their drinking? | 563 | 36.59% | 34.46% | 42.19% |
| E1h | sexual | Were you forced or pressured into sex or something sexual because of their drinking? | 568 | 5.11% | 6.50% | 3.05% |
| E1i | sococc | Did they negatively affect a social occasion you were at because of their drinking? | 567 | 58.73% | 58.76% | 59.18% |
| E1j | finances | Have you had financial trouble because of someone else's drinking? | 568 | 14.26% | 13.24% | 15.82% |
| E1k | famprob | Did you have family problems or relationship difficulties due to someone else's drinking? | 563 | 42.63% | 40.00% | 46.43% |
| E1m | caracc | Have you been involved in a traffic accident | 569 | 1.23% | 1.41% | 1.02% |
| E1n | damhouse | Was your house or other property damaged because of the drinking of any of the fairly heavy drinkers in your life (excluding the car accident we've just discussed)? | 567 | 10.93% | 10.20% | 12.18% |
| E1o | breakstuff | (Apart from damaging your house or other property) did any of the fairly heavy drinkers in your life ruin your clothes, or break or damage something that mattered to you because of their drinking? | 569 | 8.61% | 9.01% | 8.12% |

**Figure S1.** Dose-response relationship of alcohol consumption (mean weekly consumption [right panel] and frequency of heavy episodic drinking [left panel]) with the number of harms experienced

**Figure S2.** Dose-response relationship between alcohol consumption [heavy episodic drinking (left panel) and frequency of weekly consumption volume (right panel)] and number of harms experienced by gender of respondents

**Figure S3.** Dose-response relationship between alcohol consumption [heavy episodic drinking (left panel) and weekly consumption volume (right panel) and frequency] by three age groups of respondents

**Figure S4.** Dose-response relationship between alcohol consumption [weekly consumption volume (left panel) and frequency of heavy episodic drinking (right panel)] by the relationship with the most harmful heavy drinker.

**B**

**A**

**C**

**D**

**Figure S5.** Dose-response relationship between alcohol consumption [frequency of heavy episodic drinking (panels A (χ²(1) = .02, p=.900) & C (χ²(1) = .00, p=.975)) and weekly consumption volume (panels B (χ²(1) = .08, p=.778) & D (χ²(1) = .17, p=.684))] and alcohol-related harm experience [harm rates (panels A & B) and number of harm items experienced (panels C & D)] by the socioeconomic status (SEIFA index) of respondents

**B**

**D**

**C**

**A**

**Figure S6.** Dose-response relationship between alcohol consumption [frequency of heavy episodic drinking (panels A & C) and weekly consumption volume (panels B & D)] and alcohol-related harm experience [harm rates (panels A (C (χ²(1) = 1.54, p=.215) & B (C (χ²(1) = 0.10, p=.750)) and number of harm items experienced (panels C (χ²(1) = 2.98. p=.084) & D (χ²(1) = 3.53. p=.060))] by the region (urban vs rural) of respondents

**Table S2 Model outputs for number of types of harm (harm items) experienced and frequency of HED**

**Table S3 Model outputs for number of harm (harm items) experienced and weekly alcohol consumption**
